# Dual PDK1/Aurora Kinase A Inhibitors Reduce Pancreatic Cancer Cell Proliferation and Colony Formation

**DOI:** 10.3390/cancers11111695

**Published:** 2019-10-31

**Authors:** Ilaria Casari, Alice Domenichini, Simona Sestito, Emily Capone, Gianluca Sala, Simona Rapposelli, Marco Falasca

**Affiliations:** 1 Metabolic Signalling Group, School of Pharmacy and Biomedical Sciences, Curtin Health Innovation Research Institute, Curtin University, Bentley 6102, Australia; ilaria.casari@curtin.edu.au (I.C.); alice.domenchini@curtin.edu.au (A.D.); 2 Department of Pharmacy, University of Pisa, Via Bonanno, 6, 56126 Pisa, Italy; simona.sestito@for.unipi.it (S.S.); simona.rapposelli@unipi.it (S.R.); 3 Dipartimento di Scienze Mediche, Orali e Biotecnologiche, University “G. d’Annunzio” di Chieti-Pescara, Center for Advanced Studies and Technology (CAST), 66100 Chieti, Italy; caponemily@gmail.com (E.C.); g.sala@unich.it (G.S.)

**Keywords:** 3-phosphoinositide-dependent protein kinase 1, aurora kinase, PDK1/aurora A inhibitor, dual inhibitor, pancreatic cancer, cell proliferation, cell migration

## Abstract

Deregulation of different intracellular signaling pathways is a common feature in cancer. Numerous studies indicate that persistent activation of the phosphoinositide 3-kinase (PI3K) pathway is often observed in cancer cells. 3-phosphoinositide dependent protein kinase-1 (PDK1), a transducer protein that functions downstream of PI3K, is responsible for the regulation of cell proliferation and migration and it also has been found to play a key role in different cancers, pancreatic and breast cancer amongst others. As PI3K is being described to be aberrantly expressed in several cancer types, designing inhibitors targeting various downstream molecules of PI3K has been the focus of anticancer agent development for a long time. In particular, dual inhibitory drugs targeting key signaling molecules in the PI3K pathway have attracted the attention of scientists. Several drugs have progressed to clinical trials, with limited success due to toxicity and bioavailability concerns. Very few anticancer drugs targeting the PI3K pathway have been approved for clinical use and their efficacy is particularly limited towards certain tumors such as pancreatic cancer. Here, we tested two drugs displaying dual inhibitory activity towards PDK1 and Aurora kinase A in a panel of pancreatic cancer cell lines and in two in vivo models of pancreatic cancer. Our data show that both inhibitors are able to impair cell proliferation and clonogenic potential in pancreatic cancer cells. However, the limited activity of both compounds in vivo indicates that further optimization of the pharmacokinetics properties is required.

## 1. Introduction

Numerous investigations aimed at unravelling the mechanisms involved in tumorigenesis have identified fundamental cell signaling pathways implicated in cancer development. One of these key regulators, responsible for controlling the processes involved in cells metabolism, survival, and proliferation, is the intracellular phosphoinositide 3-kinase/protein kinase-B/mechanistic target of rapamycin (PI3K/Akt/mTOR) signaling pathway, that has been discovered to be dysregulated in several types of cancer [1]. The persistent activation of PI3K/Akt/mTOR pathway is known to be involved in tumor development and resistance to anticancer therapies [2].

The highly conserved PI3K pathway is stimulated by receptor tyrosine kinases and G protein-coupled receptors leading to the phosphorylation of the 3′ position of the inositol ring of phosphoinositides to activate downstream targets such as the 3-phosphoinositide-dependent kinase 1 (PDK1) [3]. At the plasma membrane, PDK1 phosphorylates and partially activates Akt at Threonine 308 (Thr308) [4]. Full activation of Akt is mediated by mTOR complex 2 (mTORC2), through phosphorylation at serine 473 (Ser473) [5]. Once activated, Akt regulates several downstream targets that mediate key cellular functions such as cell growth and survival [6].

Another crucial PI3K pathway transducer is the master kinase PDK1 that activates Akt, ribosomal protein S6 kinase beta-1, serum- and glucocorticoid-regulated kinase, ribosomal S6 kinase, and protein Kinase C isoforms ubiquitously [7]. Akt regulates many downstream effectors such as tuberous sclerosis complex 2 (TSC2) and proline-rich Akt substrate (PRAS40), which in turn regulate mTORC1 activation, a key player in protein synthesis and cell growth [8]. Additionally, Akt also controls forkhead transcription factor 3a, Bcl-2 associated death promoter, glycogen synthase kinase-3β, or mouse double minute 2 homolog. Overall, Akt-dependent pathways control several cellular functions including cell survival [5]. Therefore, the inhibition of Akt and PDK1 is considered a prominent strategy in tumor settings associated with hyperactivation of the PI3K/mTOR pathway [9,10]. We have recently found that treatment with PDK1-specific inhibitors decreased anchorage-dependent and anchorage-independent growth of pancreatic cancer cell lines, and pancreatic tumor growth in a xenograft model [11].

Aurora kinase A is a serine/threonine kinase implicated in cell mitosis and regulation of cell proliferation [12]. Aurora A is frequently overexpressed in several cancers including pancreatic cancer and its inhibition impairs cancer cell proliferation [13]. Several Aurora kinase inhibitors have been developed and introduced in cancer treatment [12,14,15]. However, their clinical utilization encountered several issues, such as side effects and drug resistance [15]. Therefore, it is imperative to have novel molecules with different chemical structures and mechanisms of action. Interestingly, higher response rates against hematologic malignancies were observed when the Aurora inhibitor showed a dual inhibitory activity such as an additional inhibitory activity towards another oncogenic driver [16]. Hence, concurrent inhibition of a cancer-inducing gene and Aurora kinases using a combination of selective Aurora inhibitors and a selective oncogene inhibitor, or utilizing compounds with dual inhibitory activity, has been proposed as a strategy to attain a better clinical outcome and overcome chemotherapy resistance [15].

Several studies have been focused on targeting different molecules of both PI3K and Aurora kinase pathways. In this study, two novel dual inhibitors targeting PDK1 and Aurora kinase A have been tested in a panel of pancreatic cancer cell lines and compared to non-malignant cells to check the effect of their activity on PI3K and Aurora kinase A pathways, and their potential as anticancer agents.

## 2. Results

### 2.1. In Vitro Kinase Profiling of Dual PDK1/Aurora Kinase Inhibitors

Both PDK1 and Aurora kinase are two emerging targets in pancreatic cancer [17,18]. Therefore, we decided to characterize the preclinical activity of our recently synthesized double PDK1/Aurora kinase inhibitors on pancreatic cancer, namely SA16 and IB35 [19]. We have previously carried out a protein kinase activity screen for SA16 and IB35 (SelectScreen Kinase Profiling Service, Invitrogen-Life Technologies). Among almost 60 protein kinases screened, both SA16 and IB35 (500 nM) displayed a very high inhibitory activity towards PDK1 and Aurora kinase A and they did not affect (percentage of inhibition <20%) any of all the other tested kinases [19,20]. Furthermore, results from SelectScreen Kinase Profiling Service (Invitrogen-Life Technologies) 10-point titration showed that the IC_50_s of SA16 and IB35 towards PDK1 were 416 nM and 112 nM respectively, whereas the corresponding IC_50_s toward Aurora kinase A were 39 nM and 289 nM, respectively [19,20]. These data clearly indicate that SA16 and IB35 are novel, potent, and highly selective PDK1/Aurora kinase A dual inhibitors. To determine whether the inhibitors were selective towards Aurora kinase A or whether they could also affect the activity of Aurora kinase B, the Kinase-Glo^®^ Luminescent Kinase Assay Platform (Promega Corp) was used. The assay was specifically designed to analyze SA16 and IB35 inhibitory effect on Aurora kinase B in a cell-free environment. Results show that the percentage of inhibition of Aurora kinase B is very low. Specifically, SA16 and IB35, used at a concentration of 10 µM, only inhibited Aurora kinase B by 9.01% ± 5.25 and 5.70% ± 4.04, respectively, compared to no substrate (Ctrl) (Supplementary Figure S1).

### 2.2. Dual Aurora Kinase/PDK1 Inhibitors Affect Pancreatic Cancer Cell Proliferation

**Figure 1:**
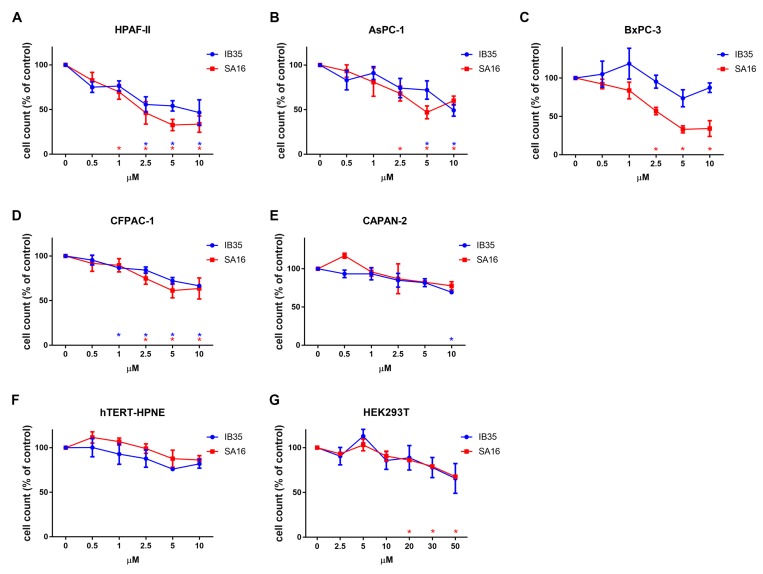
Dual 3-phosphoinositide-dependent kinase 1 (PDK1)/Aurora A inhibitors reduce pancreatic cancer cell number. HPAF-II (**A**), AsPC-1 (**B**), BxPC-3 (**C**), CFPAC-1 (**D**), CAPAN-2 (**E**), hTERT-HPNE (**F**), and HEK293T (**G**) cells were treated with the indicated concentrations of SA16 and IB35 and the number of cells was assessed after 72 h. Control cells were incubated with vehicle (DMSO, Sigma Aldrich, St. Louis, MO, USA) alone (“control”). Data are expressed as percentage of control cells and are means ± SEM of *n* = 3 independent experiments performed in duplicate. * in red: SA16; * in blue: IB35.

We next determined the effect of the dual PDK1/Aurora Kinase A inhibitors on cell growth using a panel of five human pancreatic cancer cell lines displaying distinct genetic complexity, and on one non-malignant human pancreatic duct cell line (hTERT-HPNE). Interestingly, treatment with low micromolar (1–10 μM) concentrations of either inhibitors for 72 h was able to reduce the number of HPAF-II cells (Figure 1A), with an IC50 of 2.94 μM for SA16 and 3.8 μM for IB35. One-way ANOVA was used to confirm that SA16 significantly reduced HPAF-II cell number (F_(5, 11)_ = 9.563, *p* = 0.0010) when used at concentrations between 1 and 10 μM. Similarly, IB35 effect was significant (F_(5, 12)_ = 4.068, *p* = 0.0079) at concentrations between 2.5 and 10 μM. Treatment in AsPC-1 (Figure 1B) and BxPC-3 (Figure 1C) cells showed that SA16 has a very similar inhibitory activity compared to HPAF-II, whereas IB35 showed a lower activity. Treatment with SA16 showed a significant reduction of cell number in AsPC-1 (F_(5, 12)_ = 4.641, *p* = 0.0160) and BxPC-3 (F_(5, 12)_ = 16.96, *p* < 0.0001) at 2.5, 5, and 10 μM. Conversely, treatment with IB35 was significantly effective in AsPC-1 (F_(5, 12)_ = 4.068, p = 0.0216) only at 5 and 10 μM, whereas no significant effect was reported in BXPC-3 cells (F_(5, 12)_ = 1.536, *p* = 0.2509). In CFPAC-1 (Figure 1D), the two inhibitors showed a similar activity but less pronounced compared to that observed in HPAF-II cells. Treatment with IB35 significantly reduced the number of CFPAC-1 cells (F_(5, 12)_ = 14.52, *p* < 0.0001) at all concentrations between 1 and 10 μM, while SA16 showed a significant effect (F_(5, 12)_ = 3.958, *p* = 0.0236) at 2.5, 5 and 10 μM. In CAPAN-2 (Figure 1E) cells, no significant effect was observed overall after treatment with SA16 (F_(5, 6)_ = 2.692, *p* = 0.1299) or IB35 (F_(5, 12)_ = 3.079, *p* = 0.0513), the latter only showing an effect at 10 μM (*p* = 0.0059). No significant inhibition on cell growth (for IB35, F_(5, 12)_ = 1.586, *p* = 0.2372 and for SA16, F_(5, 12)_ = 3.032, *p* = 0.0536) was observed in the non-malignant pancreatic duct cell line hTERT-HPNE (Figure 1F). Similarly, when we treated another non-malignant cell line, the human embryonic kidney cells HEK293T, with increasing concentrations (up to 50 μM) of either compounds, we observed no statistically significant effect of IB35 (F_(6, 14)_ = 2.098, *p* = 0.1188) and a significant effect of SA16 (F_(6, 15)_ = 14.68, *p* < 0.0001) only when used at the highest concentrations (20 μM and above) tested (Figure 1G).

### 2.3. Dual Aurora Kinase/PDK1 Inhibitors Reduce Anchorage Independent Pancreatic Cancer Cell Growth

**Figure 2:**
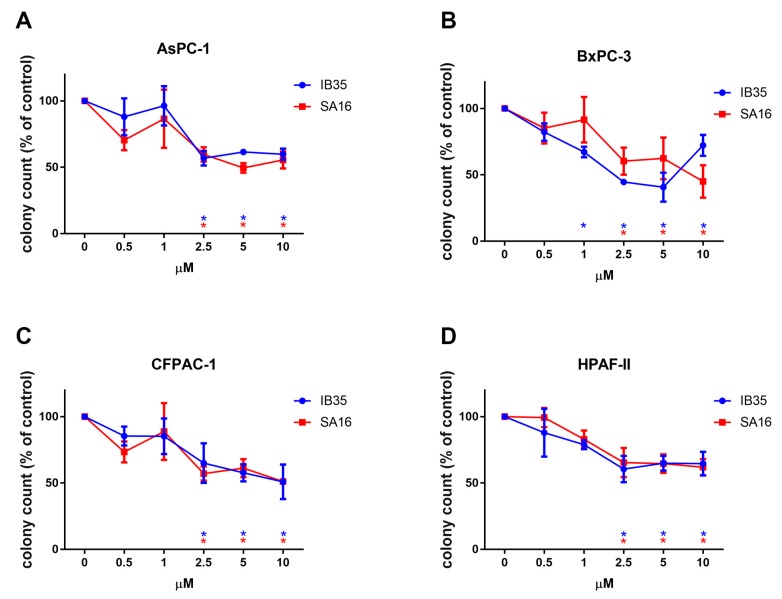
Dual PDK1/Aurora A inhibitors reduce pancreatic cancer anchorage-independent colonies formation (3D assay). AsPC-1 (**A**), HPAF-II (**B**), CFPAC-1 (**C**), and BxPC-3 (**D**) cells were treated with the indicated concentrations of SA16 and IB35 and the number of colonies was assessed after 28 days. Untreated cells were incubated with the corresponding amount of vehicle (“control”). Data are expressed as percentage of control cells and are means ± SEM of at least *n* = 3 independent experiments performed in duplicate. * in red: SA16; * in blue: IB35.

We next tested the antitumorigenic activity of the dual PDK1/Aurora kinase inhibitors in vitro by investigating their effect on anchorage-independent growth (3D growth) of pancreatic cancer cells. These properties of cancer cells are assumed to reflect the capacity of tumor cells to survive and proliferate in the harsh conditions that they face in vivo. As shown in Figure 2, both compounds were able to inhibit colonies formation of AsPC-1 (Figure 2A), BxPC-3 (Figure 2B), CFPAC-1 (Figure 2C), and HPAF-II (Figure 2D) cell lines, with each inhibitor significantly and dose-dependently reducing the number of colonies, as assessed by soft agar assays. For each treatment, and for each cell line, we performed a one-way ANOVA to compare the effect of the dose response. In AsPC-1, the number of colonies was significantly reduced upon treatment with concentration of 2.5, 5, and 10 μM of either IB35 (F_(5, 18)_ = 5.039, *p* = 0.0046) or SA16 (F_(5, 18)_ = 3.662, *p* = 0.0184).

In BxPC-3, IB35 treatment significantly reduced anchorage-independent growth at 1, 2.5, 5, and 10 μM doses (F_(5, 12)_ = 12.46, *p* = 0.0002), while SA16 was effective at 2.5, 5, and 10 μM (F_(5, 18)_ = 2.921, *p* = 0.0421). Similarly, in CFPAC-1, IB35 (F_(5, 18)_ = 3.231, *p* = 0.0296), and SA16 (F_(5, 18)_ = 3.659, *p* = 0.0185) treatments reduced colony growth in soft agar assay significantly at 2.5, 5 and 10 μM. Finally, in HPAF-II, anchorage-independent growth was significantly reduced by treatment with IB35 (F_(5, 16)_ = 4.904, *p* = 0.0065) and SA16 (F_(5, 18)_ = 6.065, *p* = 0.001) at 2.5, 5, and 10 μM.

### 2.4. Dual Aurora Kinase/PDK1 Inhibitors Inhibit Pancreatic Cancer Cell Migration

**Figure 3:**
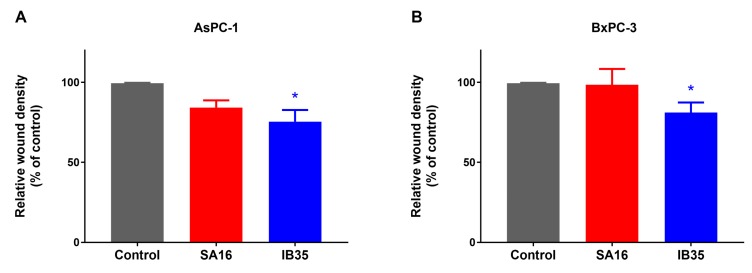
Cell migration of AsPC-1 (**A**) and BxPC-3 (**B**) cells treated with 10 μM SA16 and IB35 was assessed by wound healing assay 24 h post-wound. Control cells were treated with vehicle alone (DMSO). Wound density was determined at 24 h post wound using IncuCyte ZOOM™ assay data (* *p* < 0.05 versus control, Student’s *t* test). Data are expressed as percentage of control cells and are means ± SEM of at least *n* = 3 independent experiments.

Several studies, including our own, have previously shown that PDK1 plays a key role in cell migration [7,21,22]. Therefore, we tested the ability of SA16 and IB35 to impair cell migration in a wound healing scratch assay. Interestingly IB35, but not SA16, was able to inhibit cell migration in AsPC1-1 (Figure 3A) and BxPC-3 (Figure 3B) cells. A representative image of a wound healing assay performed in BxPC-3 cells is shown in Supplementary Figure S2.

### 2.5. Functional Validation of Dual Aurora Kinase/PDK1 Inhibitors

**Figure 4:**
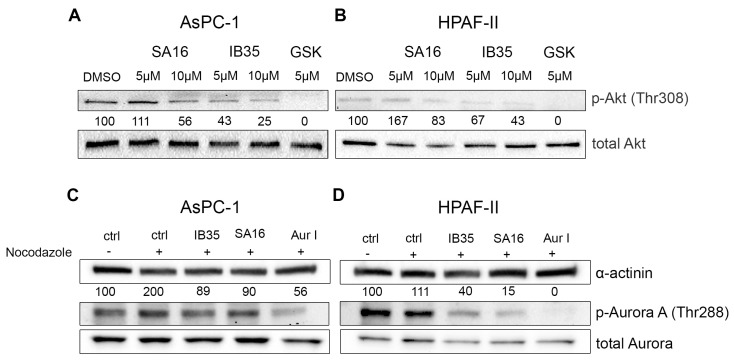
Effect of SA16 and IB35 on signaling pathways in pancreatic cancer cell lines. AsPC-1 (**A**) and HPAF-II (**B**) were serum starved for 24 h and then stimulated with FBS (DMEM + 10% FBS) for 1 h in the presence or absence of SA16, IB35, and GSK2334470 (5 μΜ) prior to cell lysis. AsPC-1 (**C**) and HPAF-II (**D**) were treated with nocodazole for 24 h and then treated with SA16, IB35, or Aurora-A Inhibitor I (10 µM) for 1 h prior to cell lysis. Lysates were then assessed by Western blotting using the indicated antibodies. Representative blots of at least three independent experiments are shown. In all blots, Akt total (**A**,**B**) and α-actinin (**C**,**D**) were used as loading control. In (**A**,**B**) membranes incubated with pAkt (Thr308) were stripped and re-incubated with anti Akt. The percentage of phosphorylated Akt is calculated by normalization to the loading control total Akt. In (**C**,**D**) membranes incubated with pAurora (Thr288) were stripped and re-incubated with anti-Aurora A. The percentage of phosphorylated Aurora A is calculated by normalization to the loading control α-actinin.

In order to gain insight into the mechanism responsible for the detected cell growth inhibition, we next determined the effect of the dual inhibitors on specific signaling pathways activation. First, we found that treatment of AsPC-1 (Figure 4A) and HPAF-II (Figure 4B) with either SA16 or IB35, when used at 10 µM, impaired the FBS-induced phosphorylation of Akt at its residue Thr308, a bona fide readout of PDK1 activity, with no effect on the total levels of Akt. In the same experimental conditions, treatment with the PDK1 inhibitor GSK2334470 also efficiently reduced Akt phosphorylation at Thr308. In parallel, we investigated the ability of SA16 and IB35 to inhibit Aurora kinase A phosphorylation at the Thr288 site in pancreatic cancer cell lines synchronized with nocodazole (200 ng/mL). In both AsPC-1 (Figure 4C) and HPAF-II (Figure 4D), SA16 and IB35, at a concentration of 10 µM, inhibited the phosphorylation of Aurora A at the residue Thr288. In the same experimental conditions, treatment with the Aurora kinase A-specific inhibitor Aur I also efficiently reduced Aurora A phosphorylation at Thr288.

**Figure 5:**
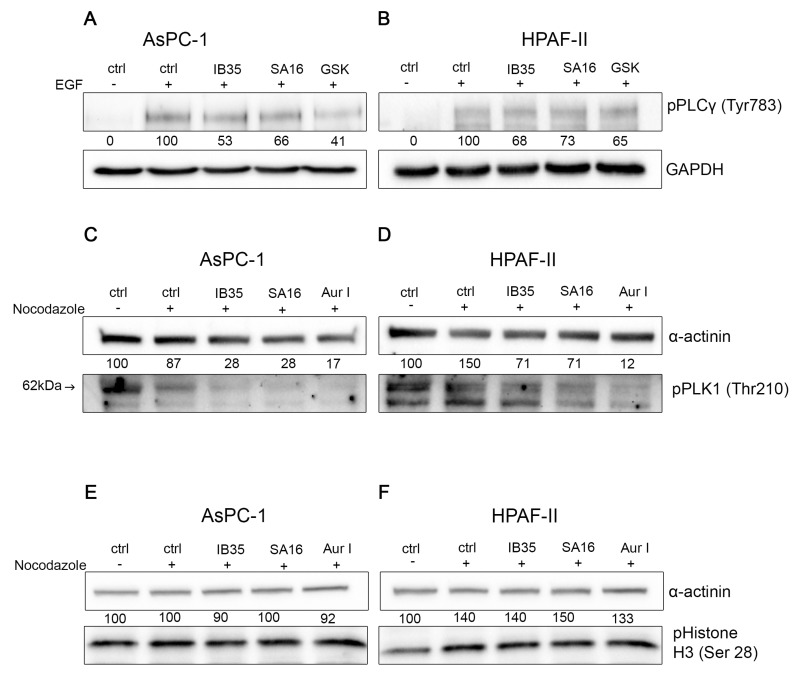
(**A**,**B**) Effect of SA16 and IB35 on PLCγ1 Tyr783 phosphorylation. AsPC-1 (**A**) and HPAF-II (**B**) were serum starved for 24 h, treated for 30 min with IB35, SA16 (both 10 μΜ) or GSK2334470 (5 μΜ) and stimulated for 10 min with 25 ng/mL of EGF to induce activation of PLCγ1. Lysates were then assessed by Western blotting using the indicated antibodies. Representative blots of at least three independent experiments are shown. The percentage of phosphorylated PLCγ1 is calculated by normalization to the loading control GAPDH. (**C**,**D**) Effect of SA16 and IB35 on the activation of PLK1 at Thr210. AsPC-1 (**C**) and HPAF-II (**D**) were treated with nocodazole (200 ng/mL) 24 h, treated for 1 h with IB35, SA16 (both 10 μΜ), or Aurora A Inhibitor (5 μΜ). The percentage of phosphorylated PLK1 is calculated by normalization to the loading control α-actinin. (**E**,**F**) Effect of SA16 and IB35 on the phosphorylation of Histone H3 at the Ser28 residue. AsPC-1 (**E**) and HPAF-II (**F**) were treated with nocodazole 200 ng/mL 24 h, treated for 1 h with IB35, SA16 (both 10 μΜ), or Aurora A Inhibitor (5 μΜ). The percentage of phosphorylated Histone H3 is calculated by normalization to the loading control α-actinin.

To validate further the specific inhibition of the PDK-1 signaling pathway, we investigated the effect of SA16 and IB35 treatments on the epidermal growth factor (EGF)-induced phosphorylation of the phospholipase C (PLC) γ1 at the Tyr783 residue, which is modulated by PDK1 [21,22]. Results show that EGF stimulation induced PLCγ1 activation via phosphorylation of the Tyr783 residue in AsPC-1 cells (Figure 5A, ctrl + EGF) and in HPAF-II cells (Figure 5B, ctrl + EGF). In the presence of either IB35 or SA16 (at a concentration of 10 μM) or GSK2334470 (5 μM), the EGF-induced activation of PLCγ1 was strongly reduced. In AsPC-1 (Figure 5A), IB35 inhibited PLCγ1 phosphorylation by about 50%, while the SA16 inhibition of PLCγ1 in the presence of EGF was less marked. In HPAF-II, the two compounds reduced the activation of PLCγ1 by about 30% (Figure 5B). In addition, to verify the inhibitory activity of IB35 and SA16 towards pathway regulated by Aurora kinase, we assessed the level of phosphorylation of polo-like kinase 1 (PLK1) at Thr210 residue, which is known to be activated by Aurora A [23,24]. When cells were synchronized with nocodazole (200 ng/mL) and then treated with IB35 and SA16 both at 10 µM, alongside with the Aurora A-specific inhibitor Aur I, we observed a reduction in the phosphorylation of PLK1 (Figure 5C,D). To further verify the specificity of SA16 and IB35 toward Aurora A we have assessed the phosphorylation of histone H3 at its residue Serine 28, which is dependent on Aurora B and is sensitive to knockdown of Aurora B but not Aurora A [25,26]. As shown in Figure 5E,F neither inhibitors affected the phosphorylation of histone H3 at Ser28, the Aurora B-specific downstream substrate, confirming the in vitro data (Supplementary Figure S1) and the specificity of the compounds towards Aurora A versus Aurora B.

### 2.6. In Vivo Validation of Dual Aurora Kinase/PDK1 Inhibitors in Pancreatic Cancer Xenografts

**Figure 6:**
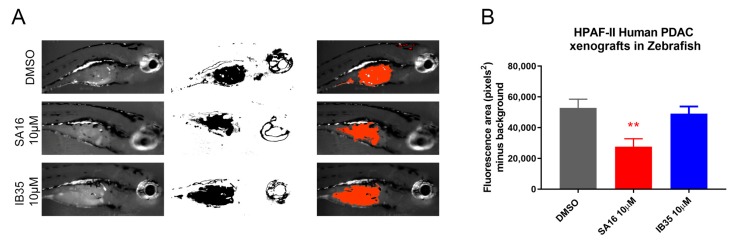
Pictures showing the tumor growth of HPAF-II xenograft zebrafish model. (**A**) The panel shows the live picture of a representative sample for each treatment vehicle (*n* = 10), 10 μM SA16 (*n* = 10) and 10 μM IB35 (*n* = 10) together with the threshold area of fluorescent-labelled cells analyzed with ImageJ and the merge of the two pictures. (**B**) Graph showing the summary of the effect on tumor burden due to the treatments. One-Way ANOVA F_(2, 23)_ = 6.709, *p* = 0.0051, multiple comparison analysis (versus DMSO) shows that SA16 significantly reduces tumor burden (*p* = 0.0044) while no effect is seen with IB35 (*p* = 0.8172). ** *p* < 0.001.

In order to evaluate the possibility to use dual PDK1/Aurora kinase A inhibitors as potential in vivo therapeutics, we performed a xenograft experiment using HPAF-II human pancreatic cancer cell line. We have decided to use the zebrafish model to establish cancer xenografts and to perform drug screening. We performed a zebrafish xenograft by implanting HPAF-II cells on 24 h post fertilization (hpf) embryos and treating them with SA16 10 μM and IB35 10 μM dissolved into the embryo medium for three days, starting from 24 h post cancer cells implantation, using the human pancreatic cancer cell line HPAF-II stained with Vibrant™-Dil dye. When analyzing the area of the fluorescence representing the Dil-labelled HPAF-II cells (Figure 6A), results showed a significant reduction in the size of the tumor burden in zebrafish treated with SA16 (Figure 6B; One-Way ANOVA, *p* = 0.044 when compared to DMSO).

**Figure 7:**
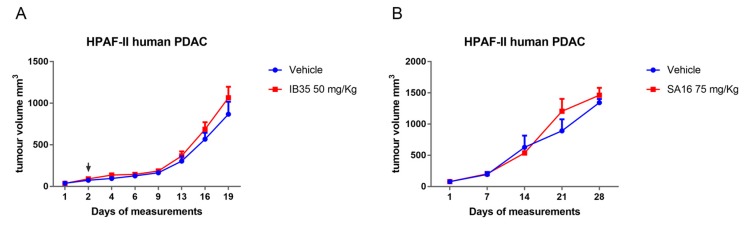
Graph showing tumor growth of HPAF-II xenograft mouse model treated with vehicle (*n* = 8) and (**A**) 50 mg/kg IB35 (*n* = 8) and (**B**) 75 mg/kg SA16 (*n* = 8). Black arrow indicates start of the treatment administration.

Considering the promising results obtained with zebrafish xenografts and a short-term treatment, we decided to perform also mice xenografts experiments using HPAF-II cells. Mice were treated with a daily intra-peritoneal dose of IB35 (50 mg/kg) and SA16 (75 mg/kg). Results showed no significant reduction of tumor volume upon treatment with either IB35 or SA16 (Figure 7A,B). A close inspection of the treated animals, after necropsy, revealed that both compounds solution had not been completely cleared but remained within the peritoneal cavity and precipitated layering on top of the internal organs, in particular liver, spleen, and intestine (Supplementary Figure S3).

## 3. Discussion

PI3K inhibitors started to make their appearance in the early 1990s. The first compounds discovered to inhibit PI3K pathway were quercetin, wortmannin and LY294002 but, even though these last two had a predominant role in the field for more than ten years, they could never be developed as pharmacological drugs to be tested in vivo due to their high level of toxicity and solubility issues [2]. Thus, scientists focused again on studying novel molecules that could target individual components of PI3K signaling pathway. Unfortunately, only few effective agents were found, such as everolimus and temsirolimus (mTORC inhibitors), and have reached approval for clinical use, even though they also presented some limitations in their employment in cancer therapies [27]. Multiple plans are being carried out with the aim of reaching clinical testing of single agents or of combinations with other chemotherapeutic drugs. Combinations of inhibitors such as rapalogs, RAD-001 and CCI-779, with chemotherapeutic drugs such as cisplatin, camptothecin, and gemcitabine are an example of the limited strategies that obtained successful results both in vivo and in vitro [28]. Some studies provided evidence that PI3K inhibition sensitizes tumors and, together with chemotherapy, presents an encouraging result in therapies. In addition, it has been demonstrated that PI3K inhibitors are well tolerated in combination with chemotherapy [29]. In an effort to inhibit distinct pathways and reduce the occurrence of drug resistance, there has been an increasing interest in the development of specific dual inhibitors.

Examples of PI3K and mTORC1 inhibitors are BEZ235, GDC-0980, and SAR245409 [30], even though bioavailability problems and high toxicity are still impairing their clinical applications. The development of inhibitors targeting the PI3K pathway has primarily focused the attention on PI3Ks and Akt, whereas PDK1 has been largely overlooked. However, the recent identification of novel pathways associated with cancer progression that are PI3K-dependent, but Akt-independent, resulted in a significant shift in this field [31]. Particularly, attention has been focused on the PDK1-mTORC2-SGK axis that has been found to substitute Akt signaling in mediating survival, migration, and growth [32]. In addition, this axis is emerging as a main process of resistance to PI3K and Akt inhibitors [33].

The compounds SA16 and IB35 were initially designed to target PDK1 and, serendipitously, found to also inhibit Aurora A [27]. SA16 and IB35 are novel dual inhibitors that could block both PDK1 and Aurora A, which are activated in several cancers.

As we recently reported that PDK1 inhibition is able to reduce pancreatic cancer cell growth, we tested IB35 and SA16 on a panel of pancreatic cancer cell lines. Our results demonstrate that both dual PDK1/Aurora A inhibitors SA16 and IB35 are able to impair cell growth and that they are not toxic at micromolar range to non-malignant human pancreatic cells and human embryonic kidney cells. The two compounds show a very similar activity but present some different results, such as the IB35 activity on cell proliferation in BxPC-3 cells and on cell migration in AsPC-1 and BxPC-3 cells. This could be explained by the different inhibitory profile possessed by SA16 that, as suggested by their in vitro IC50, inhibits Aurora A more potently than PDK1 whereas IB35 inhibits PDK1 preferentially.

This is confirmed by the fact that IB35 activity was more prominent on phosphorylation of Akt308 than SA16 and, conversely, SA16 showed a more prominent activity towards the inhibition of Aurora A T288 phosphorylation (Figure 5). To verify the specificity of IB35 and SA16 towards the inhibition of Aurora kinase A we have assessed the phosphorylation of PLK1 and Histone H3. Over a decade ago, it has been established that Aurora kinase A is responsible for the activation of PLK1 [23,24]. Indeed, our results confirm that IB35 and SA16 inhibit the phosphorylation of Aurora kinase A and, as a consequence, the phosphorylation of Thr210 of PLK1 but not the Aurora B-specific phosphorylation of serine 28 residue at Histone H3. In addition, our results show that both compounds are efficiently diminishing the EGF-induced phosphorylation of PLCγ1, with a stronger efficacy with IB35 and a less pronounced effect with SA16. These results are consistent with previous data demonstrating that EGF-induced phosphorylation of PLCγ1 is modulated by PDK1 [21,22] and confirm that IB35 is more active in inhibiting PDK1 than SA16. However, as both novel compounds provide some evidence that they are active in pancreatic cancer cell lines by targeting molecules of PI3K pathways, there is the potential for these compounds to progress into anti-cancer therapeutic agents. While it is true that PI3K signaling cascade is implicated in numerous physiological processes such as cell survival, proliferation, protein synthesis, and cellular metabolism, it has also been demonstrated that several types of cancers present aberrant expression of PI3K tumor suppressor PTEN, Akt, mTORC, and several other molecules within the PI3K/mTORC pathways. Although there are at the moment several clinical trials testing inhibitors of the PI3K pathways either as single agents or in combination with well-known chemotherapeutic drugs, there are still many issues involving their efficacy, bioavailability, specificity, and toxicity to be addressed before they can be validated into novel efficacious anti-tumor therapies [34]. Similarly, Aurora A is an emerging target in different cancers [35]. Cancer setting where both PDK1 and Aurora A are activated are those that would benefit from the development of these dual inhibitors. In this study, we have identified pancreatic cancer, one of the most aggressive malignancy, as a selected condition for further development of these agents. The results obtained from testing the two novel compounds SA16 and IB35 in cancer and non-malignant pancreatic cells suggest they could be good candidates to suppress abnormally activated molecules in both PDK1 and Aurora A signaling. Unfortunately, our in vivo assessment in mouse xenografts of both compounds revealed no activity on tumor growth, likely due to their poor pharmacokinetic properties or scarce molecular bioavailability, such as a low plasma exposure and high clearance, as previously shown for a chemical analogue in a prostate cancer xenograft [36]. Results that are more promising were obtained when the compounds were tested in zebrafish xenografts. Indeed, when dissolved in the embryo medium, there was a significant reduction in the tumor burden upon treatment with SA16, although IB35 showed no effect. The lack of activity of IB35 is possibly due to its very poor aqueous solubility and wettability compared to SA16 [37]. However, these dual inhibitors necessitate additional assessment in vivo to further clarify their mechanism of action. Further optimization to improve the pharmacokinetic and pharmacodynamic properties of our compounds are in progress, to improve the efficacy activity in order to assess their effectiveness as chemotherapeutic drugs and progress their preclinical validation. Alternatively, strategies to target tumors using nanodelivery systems could be evaluated. Another interesting aspect that warrant further investigations is to identify the pathways that are constitutively activated, or the genetic defects, in resistant versus sensitive cells that may render the cells more resistant to dual PDK1/Aurora A inhibitors. On the other hand, another possibility would be to focus on sensitive cells and identify potential biomarkers of sensitivity. For instance, we can speculate that cells resistant to both inhibitors in Figure 1 (CAPAN-2, hTERT-HPNE, and HEK293T) express all p53 wt whereas the sensitive cell lines (AsPC-1, HPAF-II, BxPC-3, and CFPAC-1) harbor a p53 mutation. Therefore, it would be interesting to investigate whether mutant p53 could be a marker of sensitivity to our PDK1/Aurora A inhibitors.

## 4. Materials and Methods

### 4.1. Inhibition of Aurora B

The inhibitory effects of IB35 and SA16 on Aurora B kinase was determined (in triplicate) using the Kinase-Glo^®^ Luminescent Kinase Assay Platform (Promega Corp.). 5 μL of compounds dissolved in 100% DMSO and DMSO as control were dispensed to each well of a 384-well plate (final concentration 10 uM) using an Echo 550 Liquid Handler. An enzyme master mix containing 1× buffer, 50 µM DTT, and 300 nM Aurora B (all reagents provided in the kit) was prepared and 2.5 μL of solution were added to each well. 2.5 μL of reaction mix containing 1× buffer, 3.33 µM adenosine triphosphate (ATP), and 7.5 ng/µL (18.75 ng/well) myelin basic protein (MBP) as substrate (buffer and MBP were provided in the Aurora B kinase Enzyme System; ultrapure ATP was acquired from Sigma Aldrich, St. Louis, MO, USA) were added to each well in order to start reaction. The plate was then sealed using thermowell sealing taper, briefly mixed, and incubated for 30 min at RT. Following this, 5 μL of Kinase-Glo^®^ Reagent were added to each well, the plate was then mixed and incubated at RT for 20 min and luminescence measurements were read using a 2300 EnSpire Multilabel reader. The luminescence signal of each sample was converted into percentage of Aurora B inhibition and compared with the average signal of no substrate (100% inhibition).

### 4.2. Cell Culture and Counting

Pancreatic cell lines AsPC-1, BxPC-3, HPAF-II, CFPAC-1, CAPAN-2, DEC-hTERT, and HEK293T were purchased from ATCC and cultured in complete growth medium supplemented with 10% FBS (Bovogen Biologicals) and 1X Penicillin-Streptomycin-Glutamine (HyClone) at 37 °C in a 5% CO_2_ atmosphere. RPMI-1640 Medium was used for AsPC-1, BXPC-3, and CAPAN-2, Eagle’s Minimum Essential Medium for HPAF-II, Iscove’s Modified Dulbecco’s Medium for CFPAC-1, and keratinocyte serum-free medium supplemented by epidermal growth factor and bovine pituitary extract (Life Technologies, Inc., Carlsbad, CA, USA) for DEC-hTERT. For the purpose of evaluating whether cell growth was affected by SA16 and IB35, 50,000 cells per well were seeded in a 12-well plate in complete medium and incubated at 37 °C in a 5% CO_2_ atmosphere for 24 h. Cells were then treated in a dose response manner and after a 72 h incubation were manually counted.

### 4.3. 3D Colony Formation

For the soft agar assay, 6-well plates were coated with 2 mL of a mixture of 1% noble agar: 2XRPMI [1:1(*v*/*v*)] incubated at 55 °C. The layer was left at room temperature (RT) until set. Cells were detached, manually counted with a Neubauer chamber and 10,000 cells per well were treated in a dose response manner in 1.5 mL mixture of warm 0.6% noble agar: 2XRPMI [1:1(*v*/*v*)] and poured on top of the coagulated first layer. After the second layer had set, each well was covered with 2 mL of 1× RPMI and plates were placed in the incubator at 37 °C in a 5% CO_2_ atmosphere for 28 days; 1× RPMI was added whenever needed. Colonies were then fixed, stained with Crystal Violet (0.05%), pictures were taken with the ChemiDoc XRS+ Imaging System from Bio-Rad Laboratories (Hercules, CA, USA) and colonies were counted.

### 4.4. Migration (Scratch Wound Assay)

80,000 cells per well from different pancreatic cancer cell lines were seeded in a 96-well plate in their suitable complete medium and incubated at 37 °C in a 5% CO_2_ atmosphere for 24 h or until 90–100% confluence was reached. Then a scratch wound was performed using the IncuCyte^®^ ZOOM Scratch WoundMaker. Cells were pre-treated with 20 μg/mL mitomycin C for 1 h at 37 °C. Cells were then treated with the inhibitors (10 μM) and placed in the IncuCyte^®^ ZOOM Live-Cell Analysis System for up to 30 h for monitoring. Pictures and movies were taken at the beginning of the process and at the time when wounds in the control wells were approximately confluent (24 h).

### 4.5. Western Blotting Analysis

Cells (300,000 per well) from different cell lines were seeded in six-well plates. The next day, five wells were stimulated with nocodazole 200 ng/mL for 24 h. Then four wells were treated for 2 h with the inhibitors of interest. To analyze the expression of phosphorylated PLCγ, cells were incubated overnight in serum-free culture media. The following day, cells were incubated with control (DMSO), or either SA16 or IB35 at 10 μM for 30 min at 37 °C, and then EGF (25 ng/mL) alone or in the presence of SA16 or IB35 10 μM was added for 10 min in serum free media. Cells were then collected by scraping and lysed in cold radioimmunoprecipitation assay buffer (150 mM sodium chloride, 1.0% NP-40 or Triton X-100, 0.5% sodium deoxycholate, 0.1% sodium dodecyl sulphate, 50 mM Tris HCl, pH 8.0) supplemented with 1X Protease/Phosphatase Inhibitor Cocktail (100X stock, Sigma Aldrich, St. Louis, MO, USA). Lysates were sonicated at 4 °C and centrifuged at 10,000 *g* for 10 min at 4 °C. Supernatants were transferred in a 1.5 mL Eppendorf tube and protein concentrations were determined using the Direct Detect Assay-Free cards and the Direct Detect Spectrometer (Merck Millipore, Decantur, IL, USA). Samples (40 μg/lane) were separated by SDS-PAGE and transferred to nitrocellulose membranes. Those were incubated in TBS containing Tween-20 (0.05% *v*/*v*) and supplemented with 3% bovine serum albumin (TBST-BSA) at RT for 1 h. Subsequently, membranes were incubated overnight at 4 °C with primary antibodies. The following day, membranes were washed with TBST at RT (three times 10 min), and incubated for 1 h at RT with the appropriate secondary antibody (1:20,000). After three washes in TBTS and two washes in PBS, membranes were incubated with Clarity Western ECL Blotting Substrates (Bio-Rad) and images were developed using the ChemiDoc XRS+ Imaging System from Bio-Rad. Specific antibodies against the following proteins were obtained from Cell Signaling; Akt (#9272), phospho-Akt (Thr308) (#4056), phospho-Aurora (Thr288) (#2914), Aurora A (#3092), pPLCγ (#2822), GAPDH (#5174), α-actinin (#3134), tubulin (#2148) phospho-PLK1 (Thr210) (#9062), phospho-Histone H3 (Ser28) (#9713). Primary antibodies were diluted in TBST-BSA (1:1000). GSK2334470 and Aurora-A Inhibitor I were obtained from Sigma Aldrich.

### 4.6. In Vivo Xenograft Experiment

Six to seven weeks old NOD/SCID (NOD.CB17-Prkdcscid/Arc) immune-deficient mice were purchased from the Animal Resources Centre (ARC-Murdoch-Western Australia) and maintained under pathogen-free conditions with water and food provided ad libitum. Mice were injected subcutaneously on either the left or the right flank with 3.5 × 10^6^ HPAF-II human pancreatic cancer cells in a volume of 250 μL of supplemented growth medium. Mice were monitored daily for tumor growth and tumor volume was measured from when it reached a palpable size using a surgical caliper. Tumor volume was determined using the equation Tumor volume = 1/2(length × width^2^). When tumors reached a volume of about 50 mm^3^, mice were randomized into two groups of nine animals per group. One group, the control group, was treated with vehicle, which is 250 μL of 0.5% carboxymethyl cellulose (CMC)/0.4% Tween-80 as a daily intra-peritoneal injection (IP). The second group, the treatment group, was treated with 250 μL of IB35 at a dose of 50 mg/kg, and SA16 at 75 mg/kg, in 0.5%CMC/0.4% Tween-80 as a daily IP injection. An unbiased operator measured tumor three times weekly. Treatment continued until the biggest tumors reached the size of 1500 mm^3^, as per Animal Ethics Guidelines. The animals’ health status was monitored daily. Procedures involving animals and their care were established according to the institutional guidelines in compliance with national and international policies. (Autorizzazione N 484/2016-PR Ministero della Salute). This research has been approved by Ministero della Salute ethic committee on 16 May 2016 (ethic code: 484/2016).

### 4.7. Zebrafish Xenograft

Wild type Tübingen (TU) zebrafish were bred and maintained in aquaria systems (Techniplast) at 28 °C; pH 7.2–7.4 and 14 h on and 10 h off light cycle as per guidelines described in the Zebrafish Book [38]. Human pancreatic cancer cell line HPAF-II at 80% confluency were stained with Vibrant™-Dil dye (ThermoFisher Scientific, Lenexa, KJ, USA). Briefly, adherent cells were rinsed once with HBSS and then incubated with Vibrant™-Dil 4 μL/mL in HBSS at 37 °C for 10 min, followed by 15 min on ice in the dark. Cells were then rinsed with PBS, harvested and resuspended at a density of 10^7^ cells/mL in cell medium. Cells were loaded into a capillary glass needle pulled using a micropipette puller (p-97 Flaming/Brown by Sutter Instrument^®^). 24 hpf zebrafish were removed from their chorion, anesthetized using MS222 and embedded in 3% methylcellulose. A volume of approximately 10 nL containing on average 100 cells was injected in the perivitelline space of each zebrafish embryo using a micromanipulator connected to a MMPI-3 pressure injector (Applied Scientific Instrumentation, Inc., Eugene, OR, USA) under a Nikon SMZ Zoom stereomicroscope. After the injection, zebrafish were gently removed from the methylcellulose and placed in a Petri dish containing fresh embryo medium to remove the effect of the anesthetic. Zebrafish were then observed under a fluorescence stereomicroscope to confirm the presence of fluorescence-labelled cells and then incubated at 34 °C O/N to allow for cell growth. The following day, 24 h post-injection, an equal number of embryos were distributed in separate wells in a 12-well plate and treated with DMSO, SA16 10 μM or IB35 10 μM respectively. At five days post fertilization (dpf), zebrafish embryos were observed and the effect of the drugs on cancer cell growth was documented using a fluorescent stereomicroscope equipped with a digital camera (Nikon, Tokio, Japan). Images were analyzed using ImageJ. Non injected embryos were used to subtract background fluorescence.

### 4.8. Statistical Analysis

Data were analyzed using GraphPad PRISM 7.0 software (La Jolla, CA, USA). Results are expressed as the mean ± standard error (SEM). One-way ANOVA with posthoc Bonferroni’s Multiple Comparison Test was used to examine the significance of the mean. T-test analysis per each measurement time point was performed for the in vivo xenograft analysis. Differences were considered significant at *p* value less than 0.05.

## 5. Conclusions

Taken together our data provide important information on the mechanisms by which two dual PDK1/Aurora kinase inhibitors impair tumor progression in pancreatic cancer models. These results support the conclusion that further preclinical studies are now needed to investigate the effect of PDK1/Aurora kinase inhibitors with improved pharmacokinetic properties and/or molecular bioavailability that might result in development of new treatments for pancreatic cancer. However, this study presents substantial limitations such as the scarce efficacy in vivo that may also reflect the notorious chemoresistance of pancreatic cancer [39]. In addition, although the in vivo efficacy of these novel dual PDK1/Aurora A kinase inhibitors appears to be overly limited by these limitations, their ability to reduce both anchorage dependent and independent growth of a panel of pancreatic cancer cell lines without affecting immortalized pancreatic cells in vitro is encouraging nonetheless. More studies therefore are now required to improve their pharmacokinetic properties in order to test their in vivo activity properly and validate them as novel potential anti-cancer compounds.

## Supplementary Materials

The following are available online at https://www.mdpi.com/2072-6694/11/11/1695/s1, Figure S1: Inhibitory effect on SA16 and IB35 on Aurora kinase B, Figure S2: Representative images of wound healing assay in BxPC-3 cells treated with SA16 and IB35 from Incucyte ZOOM^TM^ assay, Figure S3: Representative image of a xenograft NOD/SCID mouse treated with IB35 showing the compound deposition on internal organs as evidence of poor solubility and clearance. L, liver; S, spleen.

## References

[1] Fruman D.A., Chiu H., Hopkins B.D., Bagrodia S., Cantley L.C., Abraham R.T. (2017). The pi3k pathway in human disease. Cell.

[2] Falasca M. (2010). Pi3k/akt signalling pathway specific inhibitors: A novel strategy to sensitize cancer cells to anti-cancer drugs. Curr. Pharm. Des..

[3] Gagliardi P.A., Puliafito A., Primo L. (2018). Pdk1: At the crossroad of cancer signaling pathways. Semin. Cancer Biol..

[4] Mora A., Komander D., van Aalten D.M., Alessi D.R. (2004). Pdk1, the master regulator of agc kinase signal transduction. Semin. Cell Dev. Biol..

[5] Bhaskar P.T., Hay N. (2007). The two torcs and akt. Dev. Cell.

[6] Toker A., Marmiroli S. (2014). Signaling specificity in the akt pathway in biology and disease. Adv. Biol. Regul..

[7] Di Blasio L., Gagliardi P.A., Puliafito A., Primo L. (2017). Serine/threonine kinase 3-phosphoinositide-dependent protein kinase-1 (pdk1) as a key regulator of cell migration and cancer dissemination. Cancers.

[8] Manning B.D., Toker A. (2017). Akt/pkb signaling: Navigating the network. Cell.

[9] Leroux A.E., Schulze J., Biondi R.M. (2018). Agc kinases, mechanisms of regulation and innovative drug development. Semin. Cancer Biol..

[10] Raimondi C., Falasca M. (2011). Targeting pdk1 in cancer. Curr. Med. Chem..

[11] Emmanouilidi A., Fyffe C.A., Ferro R., Edling C.E., Capone E., Sestito S., Rapposelli S., Lattanzio R., Iacobelli S., Sala G. (2019). Preclinical Validation of 3-Phosphoinositide-Dependent Protein Kinase 1 Inhibition in Pancreatic Cancer. J. Exp. Clin. Cancer Res..

[12] Damodaran A.P., Vaufrey L., Gavard O., Prigent C. (2017). Aurora a kinase is a priority pharmaceutical target for the treatment of cancers. Trends Pharmacol. Sci..

[13] D’Assoro A.B., Haddad T., Galanis E. (2015). Aurora-a kinase as a promising therapeutic target in cancer. Front. Oncol..

[14] Cicenas J. (2016). The aurora kinase inhibitors in cancer research and therapy. J. Cancer Res. Clin. Oncol..

[15] Bavetsias V., Linardopoulos S. (2015). Aurora kinase inhibitors: Current status and outlook. Front. Oncol..

[16] Bavetsias V., Crumpler S., Sun C., Avery S., Atrash B., Faisal A., Moore A.S., Kosmopoulou M., Brown N., Sheldrake P.W. (2012). Optimization of imidazo [4, 5-b] pyridine-based kinase inhibitors: Identification of a dual flt3/aurora kinase inhibitor as an orally bioavailable preclinical development candidate for the treatment of acute myeloid leukemia. J. Med. Chem..

[17] Ferro R., Falasca M. (2014). Emerging role of the kras-pdk1 axis in pancreatic cancer. World J. Gastroenterol..

[18] Bearss D.J. (2011). Shining the light on aurora-a kinase as a drug target in pancreatic cancer. Mol. Cancer Ther..

[19] Daniele S., Sestito S., Pietrobono D., Giacomelli C., Chiellini G., Di Maio D., Marinelli L., Novellino E., Martini C., Rapposelli S. (2016). Dual inhibition of pdk1 and aurora kinase a: An effective strategy to induce differentiation and apoptosis of human glioblastoma multiforme stem cells. ACS Chem. Neurosci..

[20] Sestito S., Daniele S., Nesi G., Zappelli E., Di Maio D., Marinelli L., Digiacomo M., Lapucci A., Martini C., Novellino E. (2016). Locking pdk1 in dfg-out conformation through 2-oxo-indole containing molecules: Another tools to fight glioblastoma. Eur. J. Med. Chem..

[21] Raimondi C., Chikh A., Wheeler A.P., Maffucci T., Falasca M. (2012). A novel regulatory mechanism links plcγ1 to pdk1. J. Cell Sci..

[22] Raimondi C., Calleja V., Ferro R., Fantin A., Riley A.M., Potter B.V., Brennan C.H., Maffucci T., Larijani B., Falasca M. (2016). A small molecule inhibitor of pdk1/plcγ1 interaction blocks breast and melanoma cancer cell invasion. Sci. Rep..

[23] Macůrek L., Lindqvist A., Lim D., Lampson M.A., Klompmaker R., Freire R., Clouin C., Taylor S.S. (2008). Polo-like kinase-1 is activated by aurora A to promote checkpoint recovery. Nature.

[24] Seki A., Coppinger J.A., Jang C.-Y., Yates J.R., Fang G. (2008). Bora and the kinase Aurora a cooperatively activate the kinase Plk1 and control mitotic entry. Science.

[25] Goto H., Yasui Y., Nigg E.A., Inagaki M. (2002). Aurora-B phosphorylates Histone H3 at serine28 with regard to the mitotic chromosome condensation. Genes Cells.

[26] De Groot C.O., Hsia J.E., Anzola J.V., Motamedi A., Yoon M., Wong Y.L., Jenkins D., Lee H.J., Martinez M.B., Davis R.L. (2015). A Cell Biologist’s Field Guide to Aurora Kinase Inhibitors. Front. Oncol..

[27] Polivka J., Janku F. (2014). Molecular targets for cancer therapy in the pi3k/akt/mtor pathway. Pharmacol. Ther..

[28] Konings I.R., Verweij J., Wiemer E.A., Sleijfer S. (2009). The applicability of mtor inhibition in solid tumors. Curr. Cancer Drug Targets.

[29] LoRusso P.M. (2016). Inhibition of the pi3k/akt/mtor pathway in solid tumors. J. Clin. Oncol..

[30] Sabbah D.A., Brattain M.G., Zhong H. (2011). Dual inhibitors of pi3k/mtor or mtor-selective inhibitors: Which way shall we go?. Curr. Med. Chem..

[31] Sestito S., Rapposelli S. (2019). A patent update on PDK1 inhibitors (2015–present). Expert Opin. Ther. Pat..

[32] Lien E.C., Dibble C.C., Toker A. (2017). Pi3k signaling in cancer: Beyond akt. Curr. Opin. Cell Biol..

[33] Castel P., Ellis H., Bago R., Toska E., Razavi P., Carmona F.J., Kannan S., Verma C.S., Dickler M., Chandarlapaty S. (2016). Pdk1-sgk1 signaling sustains akt-independent mtorc1 activation and confers resistance to pi3kα inhibition. Cancer Cell.

[34] Alzahrani A.S. (2019). PI3K/Akt/mTOR inhibitors in cancer: At the bench and bedside. Semin. Cancer Biol..

[35] Friedberg J.W., Mahadevan D., Cebula E., Persky D., Lossos I., Agarwal A.B., Jung J., Burack R., Zhou X., Leonard E.J. (2014). Phase II study of alisertib, a selective Aurora A kinase inhibitor, in relapsed and refractory aggressive B- and T-cell non-Hodgkin lymphomas. J. Clin. Oncol..

[36] Erlanson D.A., Arndt J.W., Cancilla M.T., Cao K., Elling R.A., English N., Friedman J., Hansen S.K., Hession C., Joseph I. (2011). Discovery of a potent and highly selective pdk1 inhibitor via fragment-based drug discovery. Bioorganic Med. Chem. Lett..

[37] Kusum B., Banji D. (2010). Novel strategies for poorly water soluble drugs. Int. J. Pharm. Sci. Rev. Res..

[38] Westerfield M. (1995). The Zebrafish Book: A Guide for the Laboratory Use of Zebrafish (Brachydanio Rerio).

[39] Adamska A., Elaskalani O., Emmanouilidi A., Kim M., Razak N.B.A., Metharom P., Falasca M. (2018). Molecular and cellular mechanisms of chemoresistance in pancreatic cancer. Adv. Biol. Regul..

