# Supplementary material for: Dual PDK1/Aurora Kinase A Inhibitors Reduce Pancreatic Cancer Cell Proliferation and Colony Formation
Source: Cancers (Basel). 2019 Oct 31;11(11):1695. doi: 10.3390/cancers11111695 (PMC6896057; doi:10.3390/cancers11111695)
Supplement: Supplementary file 1 [file cancers-11-01695-s001.pdf]

Supplementary Materials: Dual PDK1/ Aurora Kinase A Inhibitors Reduce Pancreatic Cancer Cell Proliferation and Colony Formation

Ilaria Casari, Alice Domenichini, Simona Sestito, Emily Capone, Gianluca Sala, Simona Rapposelli and Marco Falasca

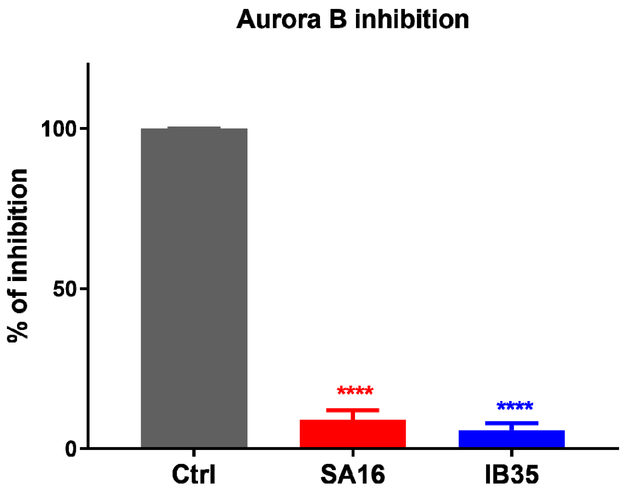

Figure S1. Graph showing the result of the Kinase-Glo® Luminescent Kinase Assay used to determine the effectiveness of SA16 and IB35 (10 μM) in inhibiting the activation of Aurora Kinase B. This is a cell-free assay and the control refers to no substrate (100% inhibition). Results are statistically significant (One-way ANOVA $F_{(2, 16)} = 3218$, $p < 0.0001$).

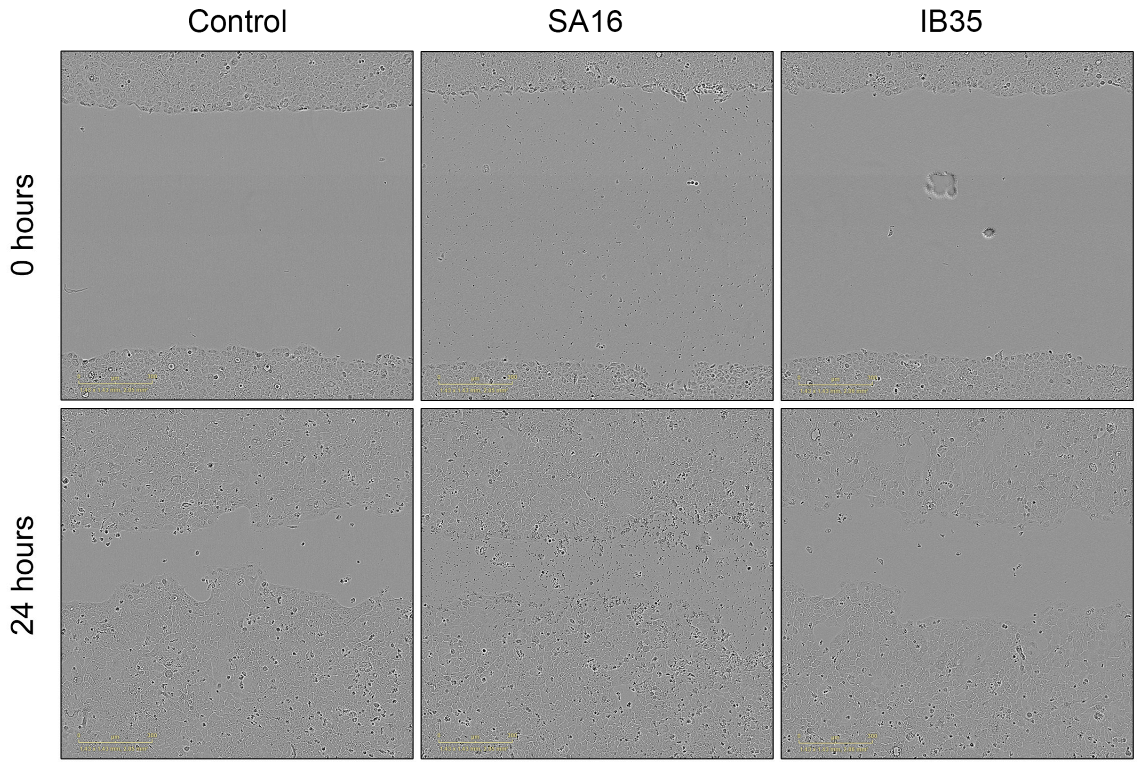

Figure S2. Representative images of wound healing assay in BxPC3 cells treated with SA16 and IB35 from Incucyte ZOOM™ assay. Scale bar = 300 µm.

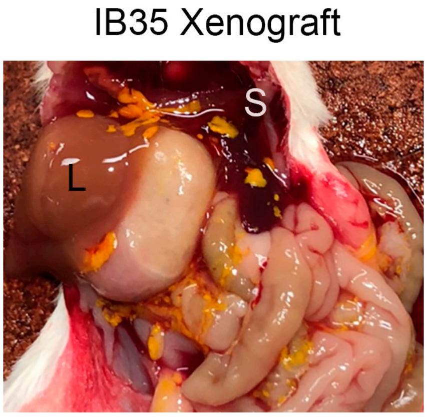

Figure S3. Representative image of a xenograft NOD/SCID mouse treated with IB35 showing the compound deposition on internal organs as evidence of poor solubility and clearance. L, liver; S, spleen.

Whole Blots

Membranes from each gel were cut at different molecular weights (corresponding to the weight of the proteins of interest) and incubated with the corresponding antibodies.

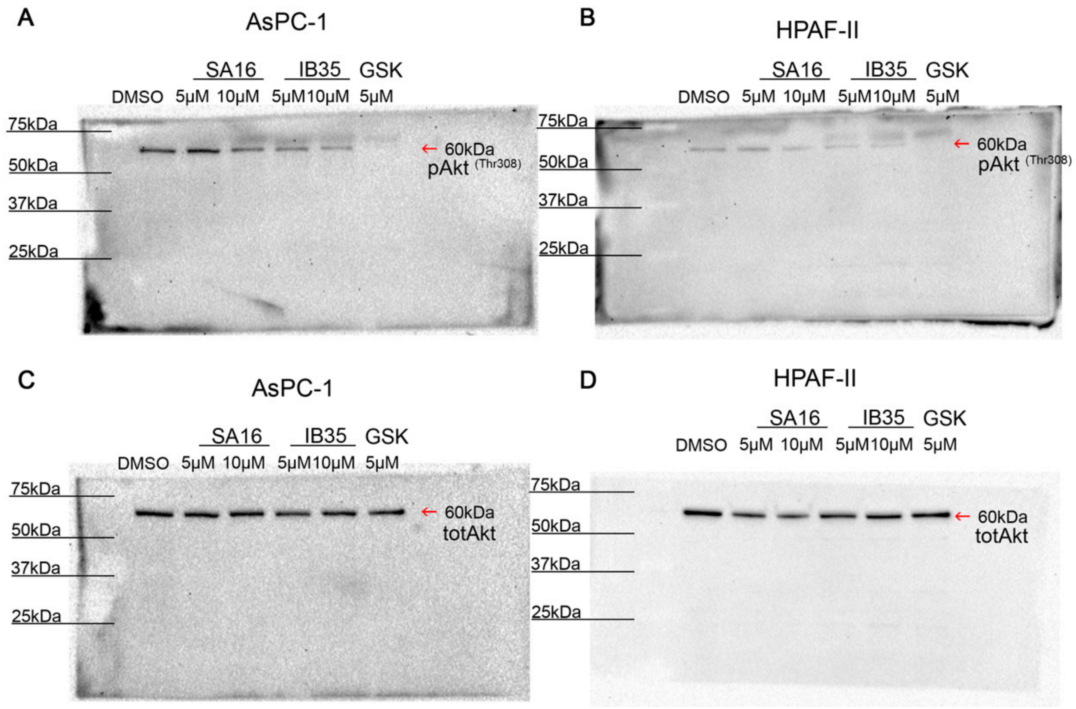

Blot 1: AsPC-1 (A) and HPAF-II (B) cell lysates probed for pAkt (Thr308). The membranes were then stripped and re-probed for total Akt as loading control. Membranes in A and B were exposed for 120 sec for an optimal signal detection while the membranes in C and D were exposed for 30 sec.

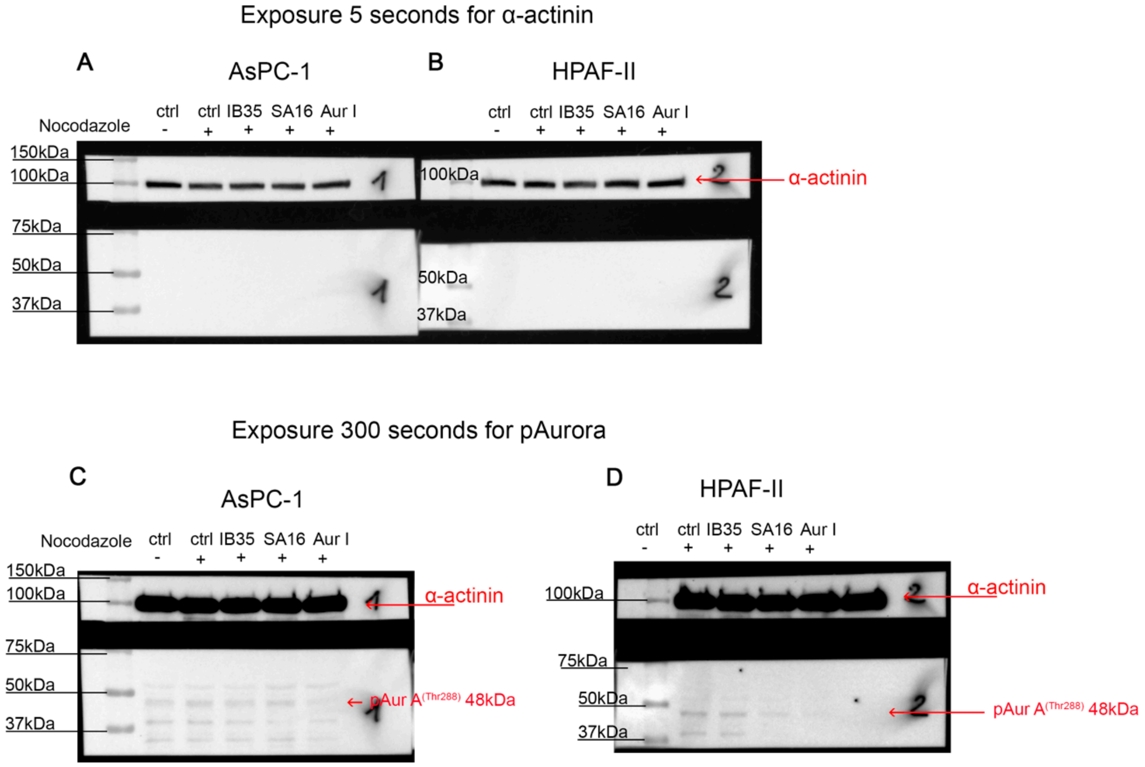

Blot 2: AsPC-1 (A) and HPAF-II (B) cell lysates probed for α-actinin as loading control and pAurora A (Thr288)/B (Thr232)/C (Thr198). Membranes in A and B were exposed for 5 as the optimised exposure time to visualise the loading control α-actinin. Five seconds of exposure time, although, was too short to visualise pAurora, which displays a weaker signal. Therefore, we performed a signal accumulation exposure and selected 300 sec as the best exposure time to visualise pAurora in both AsPC-1 (C) and HPAF-II (D).

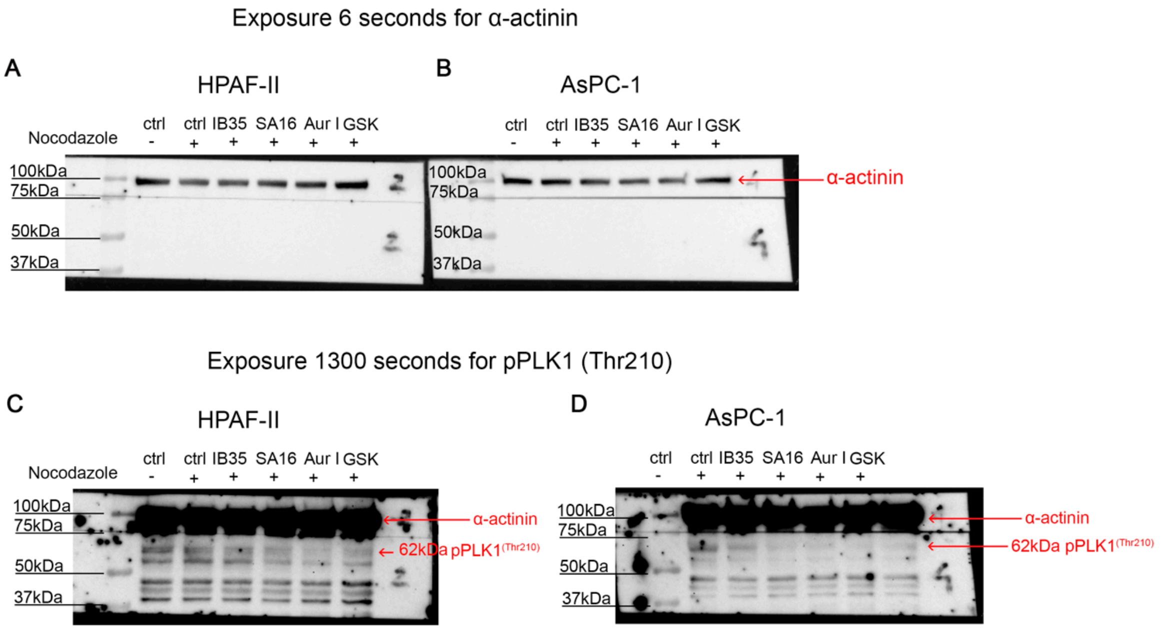

Blot 3: HPAF-II (A) and AsPC-1 (B) cell lysates probed for α-actinin as loading control and pPLK1 (Thr210. Membranes in A and B were exposed for 6 as the optimised exposure time to visualise the loading control α-actinin. Six seconds of exposure time, although, was too short to visualise pPLK1 (Thr210), which displays a weaker signal. Therefore, we performed a signal accumulation exposure and selected 1300 sec as the best exposure time to visualise pPLK1 in both HPAF-II (C) and AsPC-1 (D).

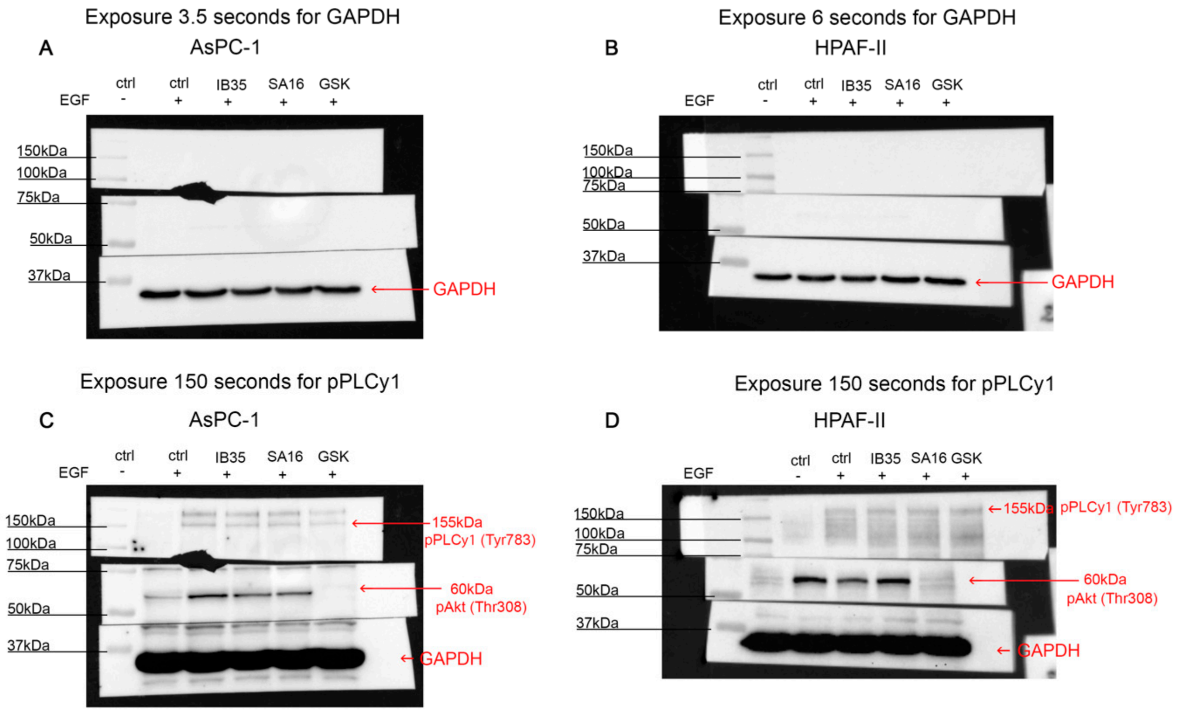

Blot 4: AsPC-1 (A) and HPAF-II (B) cell lysates probed for GAPDH as loading control, pAkt (Thr 308) used as positive control control to verify the treatment efficacy and pPLCγ (Tyr783). Membranes in A and B were exposed for 3.5 as the optimised exposure time to visualise the loading control GAPDH. This exposure time although, was too short to visualise pAkt (Thr308) and pPLCγ (Tyr783), which display a weaker signal. Therefore, we performed a signal accumulation exposure and selected 150 sec as the best exposure time to visualise pAkt (Thr308) and pPLCγ (Tyr783) in both AsPC-1 (C) and HPAF-II (D).

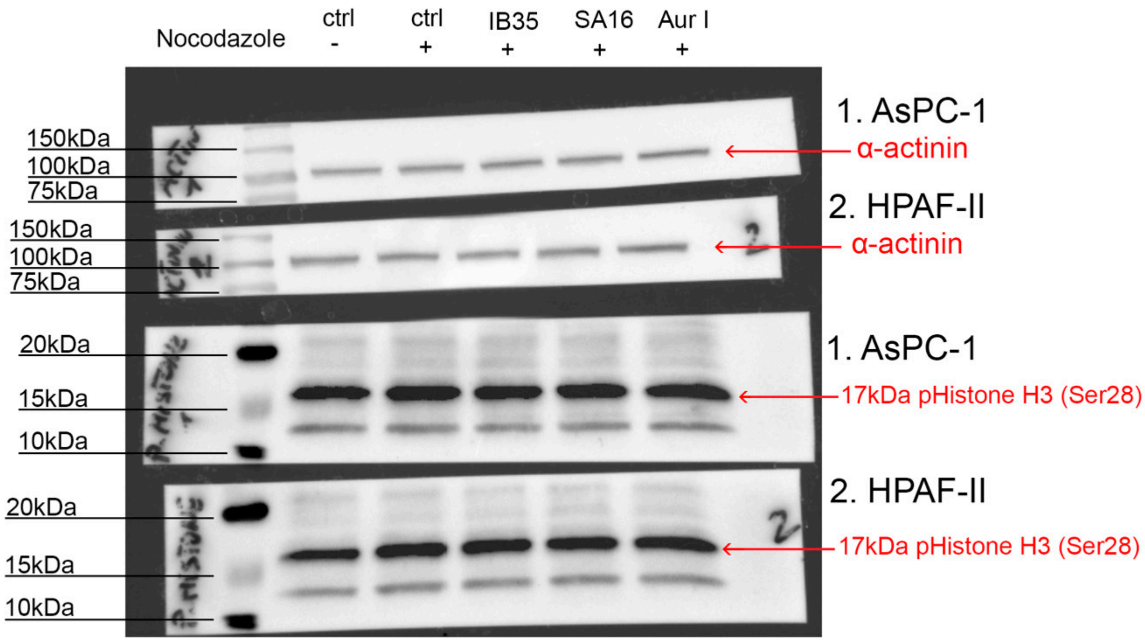

Blot 5: AsPC-1 (membrane 1) and HPAF-II (membrane 2) cell lysates probed for α-actinin as loading control and pHistone H3 (Ser28). The signal was detected with all the membranes exposed for the same exposure time of 30 sec.
